# Placental extract suppresses lipid droplet accumulation by autophagy during the differentiation of adipose-derived mesenchymal stromal/stem cells into mature adipocytes

**DOI:** 10.1186/s13104-023-06622-6

**Published:** 2023-11-16

**Authors:** Yusuke Ando, Eri Odawara, Hiroyasu Sakai, Fumiaki Sato, Junzo Kamei

**Affiliations:** 1 Laboratory of Clinical Pathology, School of Pharmacy, Faculty of Pharmacy and Pharmaceutical Sciences, Josai University, 1-1 Keyakidai, Sakado, 350-0295 Saitama Japan; 2 Laboratory for Bioanalysis and Onco-Pharmaceutics, Hoshi University School of Pharmacy and Pharmaceutical Sciences, 2-4-41 Ebara, Shinagawa-ku, Tokyo, 142-8501 Japan; 3 Department of Biomolecular Pharmacology, Hoshi University School of Pharmacy and Pharmaceutical Sciences, 2-4-41 Ebara, Shinagawa-ku, Tokyo, 1428501 Japan; 4 Juntendo Advanced Research Institute for Health Science, Juntendo University, 2-1-1 Hongo, Bunkyo-ku, Tokyo, 113-8421 Japan

**Keywords:** Placental extract, Autophagy, Lipolysis, Lysosomal acid lipase A, Adipose-derived mesenchymal stromal/stem cells, Adipogenesis

## Abstract

**Objective:**

Placental extract, which contains various bioactive compounds, has been used as traditional medicine. Many studies have demonstrated additional applications of placental extract and provided a scientific basis for the broad spectrum of its effects. We have previously reported that porcine placental extract (PPE) strongly suppresses adipogenesis in a 3T3-L1 preadipocyte cell line, inhibiting differentiation. This study aimed to examine the effect of PPE on the accumulation of lipid droplets (LD) in adipose-derived mesenchymal stromal/stem cells (ASC).

**Results:**

The study findings revealed that PPE decreased the size of LD during the differentiation of ASC into mature adipocytes. RT-qPCR analysis revealed that PPE increased the gene expression of lysosomal acid lipase A (*Lipa*), a lipolysis-related gene, in ASC-differentiated adipocytes. However, no differences were noted in the adipocyte differentiation markers (*Pparg*, *Cebpa*, and *Adipoq*), or the adipogenesis-related genes (*Dgat1*, *Dgat2*, *Fasn*, *Soat1*, and *Soat2*). In addition, PPE promoted autophagosome formation, which was partially co-localized with the LD, indicating that PPE accelerated the degradation of LD by inducing autophagy (termed lipophagy) during the differentiation of ASC into mature adipocytes. These results suggest that the use of PPE may be a potential novel treatment for regulating adipogenesis for the treatment of obesity.

**Supplementary Information:**

The online version contains supplementary material available at 10.1186/s13104-023-06622-6.

## Introduction

Obesity is currently one of the greatest global health concern, increasing the risk of diabetes, hypertension, fatty liver disease, cardiovascular disorders, and cancers [1–3]. Although the established treatments for obesity, including dietary control, exercise, surgery, and medication, have been effective, the number of overweight and obese individuals is still rapidly increasing worldwide [4, 5]. Hence, more potent treatments for controlling obesity need to be established.

The pathophysiology of obesity is influenced by genetic, environmental, and behavioral factors [6, 7]. The adipose tissue mass depends on the balance between adipogenesis and lipolysis [8], which dictates obesity. It is more complicated than previously assumed and includes epigenetic regulation [9], brown/beige adipocyte lipolysis [10], and selective autophagic degradation of lipid droplets (LD) in the lysosomes (termed lipophagy) [11, 12].

Placental extract is a traditional medicine used to treat fatigue, menopausal symptoms, skin whitening, and antiaging [13]. Since it contains various nutrients, including amino acids, nucleic acids, minerals, vitamins, hormones, and some growth factors, its applicability in treating various diseases is being explored [14, 15].

Porcine placental extract (PPE) strongly inhibits differentiation of the 3T3-L1 cell line into mature adipocytes via p38 mitogen-activated protein kinases (MAPK) activation and has therapeutic potential for treating obesity [16]. This study aimed to further explore the potential therapeutic effect of PPE on obesity by evaluating its effect on adipogenesis using adipose-derived mesenchymal stromal/stem cells (ASC) derived from mouse epididymal white adipose tissue (eWAT).

## Materials and methods

### Reagents and antibodies

Dulbecco’s Modified Eagle Medium (D-MEM)/Ham’s F-12 medium, 3-isobutyl-1-methylxanthine (IBMX), dexamethasone (DEX), and insulin were purchased from FUJIFILM Wako Pure Chemical Corporation (Osaka, Japan). Fetal bovine serum (FBS) was procured from Biosera (Boussens, France). Rosiglitazone was purchased from Tokyo Chemical Industry (Tokyo, Japan). Oil Red O was purchased from Sigma-Aldrich (MO, USA). The antibodies used in this study are listed in Additional Table 1.

### Preparation of the PPE

The PPE used in this study (Batch 131,029) was manufactured by SNOWDEN (Tokyo, Japan), using a combination of fermentation and proteolysis and was prepared as previously described [14, 16, 17].

### Differentiation of ASC into mature adipocytes

**Fig. 1:**
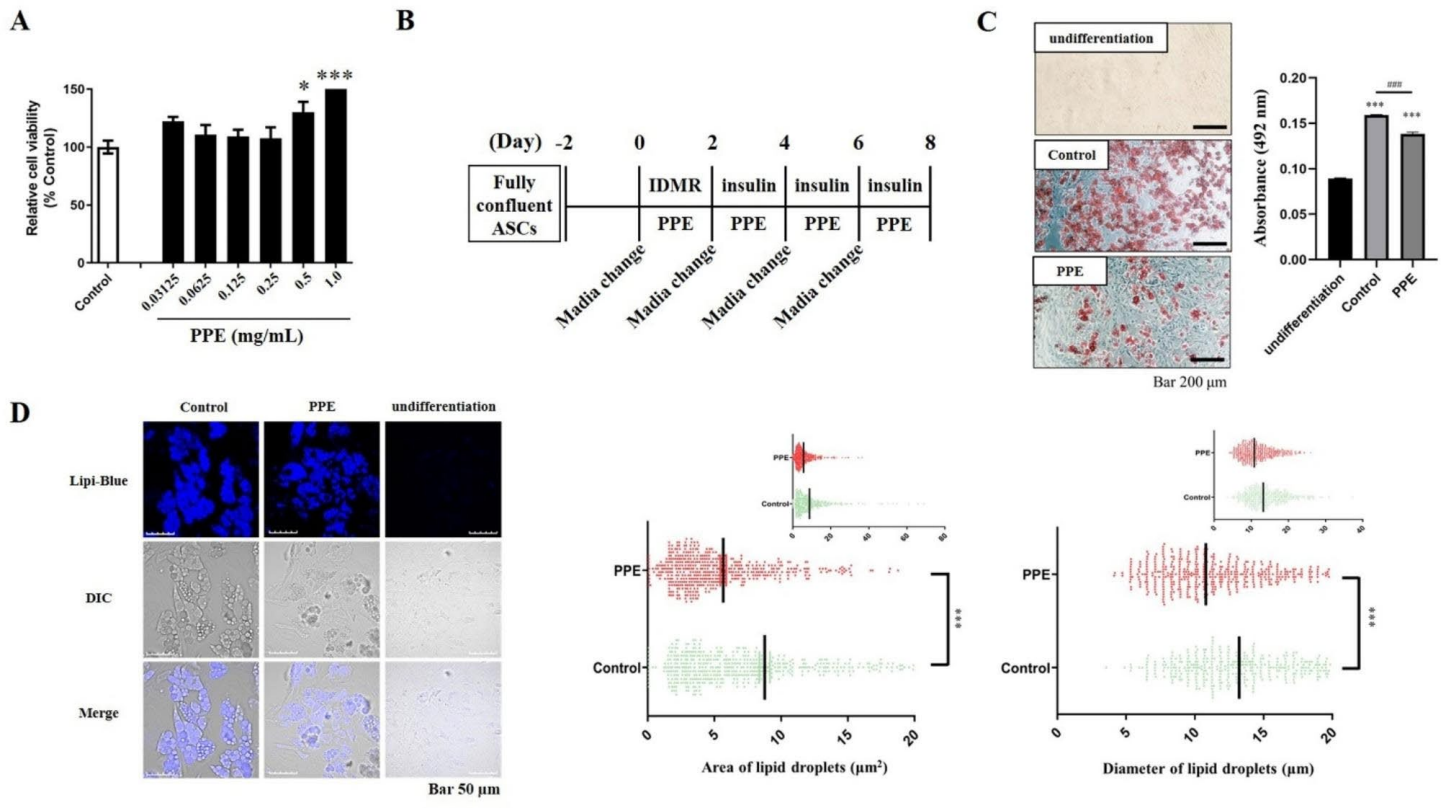
Porcine placental extract (PPE) decreased lipid droplet size in adipose-derived mesenchymal stromal/stem cells (ASC)-differentiated adipocytes (**A**) ASC were plated on a 96-well plate at a density of 1 × 10^4^ cells in complete media. After 24 h, cells were treated with various PPE concentrations (1.0, 0.5, 0.25, 0.125, 0.0625 or 0.03125 mg/mL) for additional 48 h. PPE cytotoxicity against ASC was analyzed using the WST assay (see Additional Materials and Methods). The data represent relative cell viabilities compared to ASC cultured without PPE (*Control*). Experiments were performed in triplicate, and the data are presented as the mean ± SEM. *p < 0.05, ***p < 0.001 vs. *Control*. (**B**) Schematic representation for culture with PPE during ASC differentiation. ASC reaching confluence were cultured with or without PPE for 2 days in 0.5 mM IBMX, 0.25 µM DEX, 5 µg/mL insulin, and 1 µM rosiglitazone. The medium was replaced every 2 days to fresh medium containing 5 µg/mL insulin with or without PPE until day 8, followed by staining with Oil Red O or Lipi-Blue. (**C**) LD in the ASC cultured for 8 days with or without 1.0 mg/mL PPE were stained with Oil Red O and visualized using bright field microscopy. Stained LD were extracted with isopropanol and quantified at 492 nm. Experiments were performed in triplicate, and the data are presented as the mean ± standard error of the mean (SEM) (***p < 0.001 vs. *undifferentiation*, ^###^p < 0.001 vs. *Control*). Scale bar: 200 μm. (**D**) LD in ASC cultured for 8 days with or without 1.0 mg/mL PPE were stained with Lipi-Blue fluorescent probe and visualized using a confocal laser scanning microscope; the area and diameter of cellular LD in 50 cells were quantitatively analyzed using cellSens software ver. 4.1. Each point represents a single lipid droplet, and the data are presented as the mean ± SEM. ***p < 0.001 vs. *Control*. Scale bar: 50 μm

Mouse ASC were prepared according to an established protocol [18] (see Additional Materials and Methods for details). ASC were plated on a 24-well plate at a density of 1 × 10^5^ cells/well or on an 8-well glass chamber slide (Matsunami Glass, Osaka, Japan) at a density of 1 × 10^4^ cells/well and cultured with D-MEM/Ham’s F-12 supplemented with 10% FBS (complete media) until confluence. The ASCs were further cultured with complete media for 2 days (Day 0) and then cultured with complete media containing IDMR (0.5 mM IBMX, 0.25 µM DEX, 5 µg/mL insulin, and 1 µM rosiglitazone) for 2 days. The culture medium was replaced every 2 days with complete media containing 5 µg/mL insulin and culture was continued until day 6 or day 8. The schedule for PPE culture during ASC differentiation is shown in Fig. 1B.

### Lipid droplet and autophagosome staining

Oil Red O staining was performed as previously described [16]. The Lipi-Blue and DAPRed fluorescent probes (Dojindo Laboratories) were also used to observe the LD and autophagosomes, according to the manufacturer’s protocols. Fluorescence images were obtained using an FV3000 confocal laser scanning microscope and analyzed using the cellSens software ver. 4.1 (Olympus Scientific Solutions, Tokyo, Japan).

### Reverse transcriptase-quantitative polymerase chain reaction

Total RNA was extracted from cells using the TRIzol Reagent (Thermo Fisher Scientific), and cDNA was synthesized from the extracted RNA using a ReverTra Ace qPCR RT Master Mix with gDNA Remover (TOYOBO, Osaka, Japan). The procedures were performed following the respective manufacturer’s protocols. Quantitative polymerase chain reaction (qPCR) was performed using the KAPA SYBR FAST qPCR Kit Master Mix (2X) ABI Prism (KAPA Biosystems, MA, USA). All samples were analyzed in triplicate and quantified using the relative standard curve method considering the gene expression of *Rplp0* as an internal control. The primer pairs used in this study are listed in Additional Table 2.

### Data analyses

Statistical analyses were performed using GraphPad Prism 9 (GraphPad Software, Inc., San Diego, CA). The significance of differences was determined using a two-tailed Student’s t-test, and one-way analysis of variance with Dunnet’s posthoc test. Differences were considered significant at p < 0.05. Data are the average of three samples obtained from independent experiments and values are expressed as mean ± standard error of the mean (SEM).

## Results

### PPE decreased the accumulation of LD during ASC differentiation into mature adipocytes

Initially, ASC were cultured with various PPE concentrations (0.03125–1.0 mg/mL) for 48 h to determine cytotoxicity. ASC viability remained unaltered upto 0.25 mg/mL PPE and increased thereafter (Fig. 1A). Thus, PPE was non-cytotoxic to ASC upto a concentration of 1.0 mg/mL. These results also suggest that PPE enhanced ASC proliferation at high concentrations, similar to that observed with 3T3-L1 cells [16].

We further investigated the effects of PPE on adipogenesis during ASC differentiation into mature adipocytes (Fig. 1B). PPE was non-cytotoxic to ASC during the 8 days of differentiation induction (Additional Fig. 1). Oil Red O staining (Fig. 1C, left panel) and the absorbance measurement of extracted Oil Red O (Fig. 1C, right panel) revealed that the number of LD increased with differentiation into mature adipocytes (*Control*). Similar results were obtained using a different batch of PPE (Batch 190,129) (Additional Fig. 2). The accumulation of LD was suppressed with PPE treatment during differentiation. Further, fluorescence imaging analysis revealed that PPE significantly reduced the size of LD in ASC-differentiated adipocytes (Fig. 1D).

**Fig. 2:**
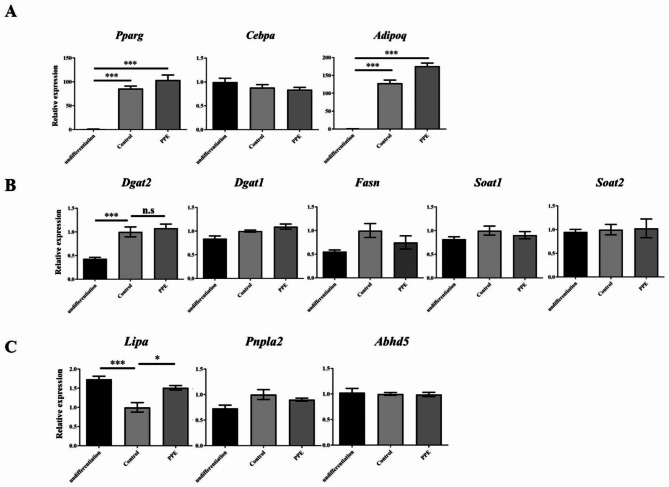
PPE induced the expression of Lipa in ASC-differentiated adipocytes The gene expression of (**A**) adipocyte differentiation markers (*Pparg*, *Cebpa*, and *Adipoq*), (**B**) adipogenesis-related genes (*Dgat1*, *Dgat2*, *Fasn*, *Soat1*, and *Soat2*), and (**C**) lipolysis-related genes (*Pnpla2*, *Abhd5*, and *Lipa*) in ASCs cultured for 8 days with 1.0 mg/mL PPE were analyzed using RT-qPCR. *Control* represents cells cultured without PPE, and *undifferentiation* represents cells cultured without PPE and differentiation-inducing agents. The gene expression of adipocyte differentiation markers is presented relative to the value of those from the *undifferentiation* cells, and the expression of adipogenesis-related genes and lipolysis-related genes are presented relative to the value in *Control*. All RT-qPCR experiments were performed in triplicate, and the data are presented as the mean ± SEM. *p < 0.05, ***p < 0.001 (A: vs. *undifferentiation*, B and C: vs. *Control*.)

### PPE increased the gene expression of lysosomal acid lipase A

We further examined the effects of PPE on the gene expression related to adipocyte differentiation in ASC. Reverse transcriptase-qPCR (RT-qPCR) analysis revealed that the expressions of *Pparg*, and *Adipoq*, but not *Cebpa*, markedly increased in ASC-differentiated adipocytes, similar to those in 3T3-L1 cells (Fig. 2A). However, PPE did not affect these gene expressions during ASC differentiation into mature adipocytes, suggesting that the decrease in lipid accumulation in ASC by PPE was independent of adipocyte differentiation.

The balance between adipogenesis and lipolysis controls the accumulation of LD [8]. Hence, we determined the expression levels of adipogenesis-(*Dgat1*, *Dgat2*, *Fasn*, *Soat1*, and *Soat2*) and lipolysis-related genes (*Pnpla2*, *Abhd5*, and *Lipa*). RT-qPCR analysis revealed that the expression levels of *Dgat2*, responsible for triacylglycerol synthesis, similar to *Dgat1* [19], significantly increased in ASC-differentiated adipocytes; however, those of *Dgat1*, *Fasn*, *Soat1*, and *Soat2* remained unchanged on day 8 (Fig. 2B). Further, PPE did not affect the expression of these genes in ASC-differentiated adipocytes.

In contrast, RT-qPCR analysis of lipolysis-related genes revealed that the expression of *Lipa*, encoding lysosomal acid lipase (LAL) A, decreased in ASC-differentiated adipocytes, but those of *Pnpla2* and *Abhd5* remained unchanged on day 8 (Fig. 2C). Interestingly, PPE increased *Lipa* expression in ASC-differentiated adipocytes or suppressed the decrease in gene expression during ACS differentiation into mature adipocytes. Thus, PPE suppressed the accumulation of LD via lipolysis with increased *Lipa* expression.

### PPE induced autophagosome formation

**Fig. 3:**
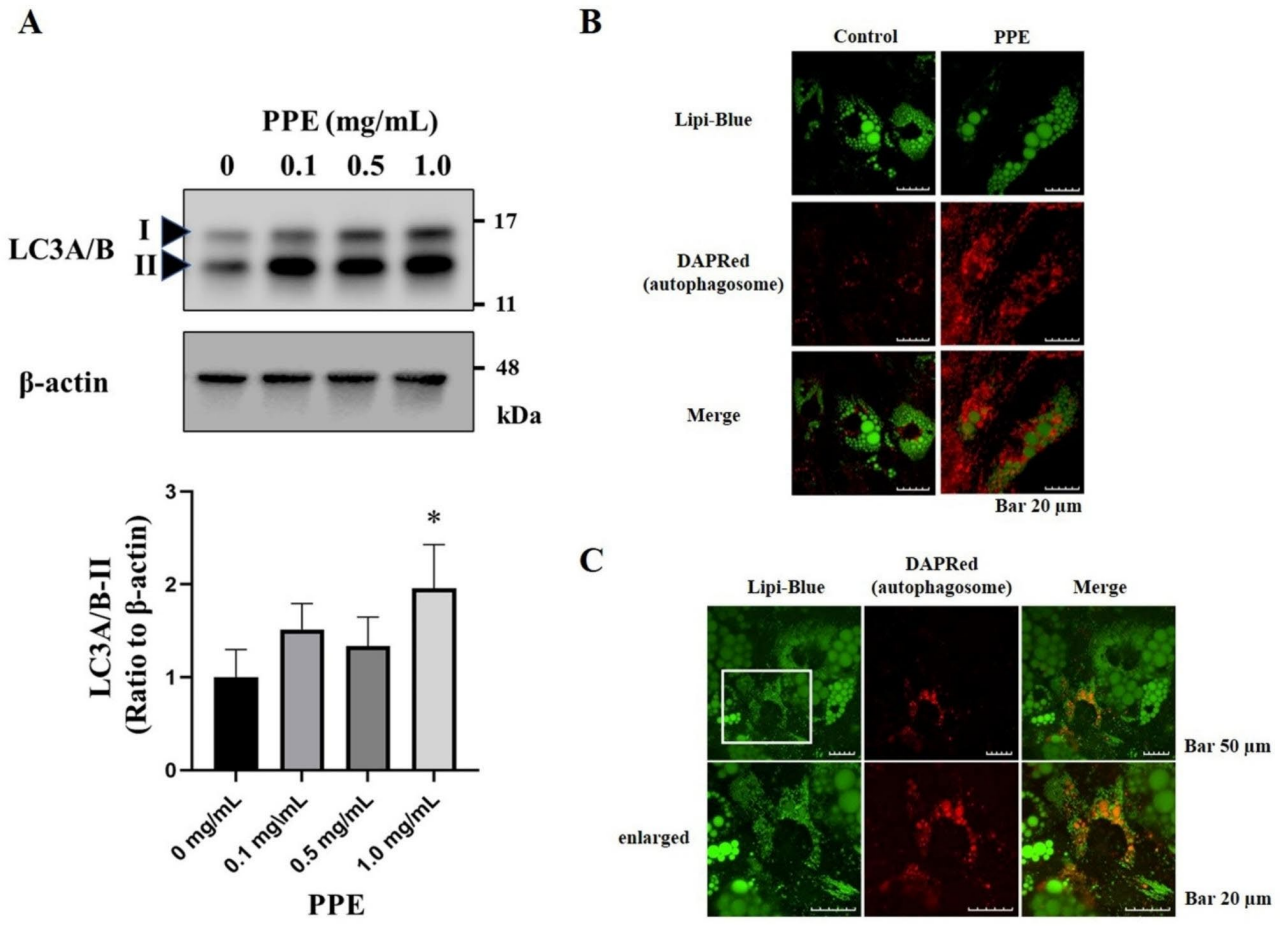
PPE facilitated autophagosome formation in ASC-differentiated adipocytes (**A**) Expression levels of LC3A/B in ASC cultured for 8 days with or without PPE (0, 0.1, 0.5 or 1.0 mg/mL) were analyzed using immunoblotting (see Additional Materials and Methods). Experiments were performed in triplicate, and the representative data are shown. Arrowheads indicate LC3A/B-I and LC3A/B-II. The relative intensity of each LC3A/B-II band after normalization to β-actin levels is shown in the lower panel. The blots were cropped, and full-length uncropped blots are presented in Additional Fig. 3. The data are presented as the mean ± standard error of the mean. *p < 0.05 vs. *0 mg/mL PPE*. (**B**) LD and autophagosomes in ASC cultured for 6 days with or without 1.0 mg/mL PPE were stained with Lipi-Blue fluorescent probe and DAPRed, and imaged using a confocal laser scanning microscope. Scale bar: 20 μm. (**C**) LD and autophagosomes in ASC cultured for 6 days with 1.0 mg/mL PPE were stained with Lipi-Blue fluorescent probe and DAPRed, and imaged using a confocal laser scanning microscope. The images in the lower panel (*enlarged*) are magnified versions of the inset marked in the upper panel. Scale bar: 50 or 20 μm (enlarged)

Lipophagy is a selective autophagy targeting LD and regulating the maintenance of LD homeostasis [12, 20, 21]. We determined the effect of PPE on lipophagy to investigate decrease in the size of accumulated LD during ASC differentiation. Immunoblotting demonstrated that PPE increased the expression levels of LC3A/B-II in ASC-differentiated adipocytes on day 8 (Fig. 3A). Similar results were obtained using a different batch of PPE (Batch 190,129) (Additional Fig. 4). Moreover, fluorescence imaging revealed that PPE enhanced autophagosome formation in ASC-differentiated adipocytes (Fig. 3B). The autophagosomes partially co-localized with the LD on day 6 (Fig. 3C), suggesting that PPE promoted lipolysis via enhancing autophagy during ASC differentiation into mature adipocytes.

## Discussion

The present study findings revealed that PPE decreased the size of LD without inhibiting adipocyte differentiation and adipogenesis during ASC differentiation into mature adipocytes. Further, PPE enhanced autophagosome formation and increased or maintained *Lipa* expression in ASC-differentiated adipocytes. Thus, PPE promoted the degradation of LD via lipophagy during ASC differentiation into mature adipocytes.

In mammalian cells, peroxisome proliferator-activated receptor γ (PPARγ) and the CCAAT/enhancer binding proteins (C/EBPs) such as C/EBPα are master transcription factors of adipogenesis in the early differentiation phase. C/EBPα cannot promote adipogenesis without PPARγ, while PPARγ can promote adipogenesis in C/EBPα-deficient cells [22–25]. In this study, the expression of PPARγ, but not C/EBPα, dramatically increased in the ASC-differentiated adipocytes. Moreover, the expression of *Adipoq*, encoding adiponectin involved in the formation of mature adipocytes, and *Dgat2*, encoding acyl-CoA: diacylglycerol acyltransferases 2, significantly increased in the ASC-differentiated adipocytes. Further, ASC differentiation resulted in LD accumulation, suggesting that C/EBPα is non-essential for ASC differentiation into mature adipocytes.

Uncoupling protein-1 (UCP1) plays an essential role in lipolysis and thermogenesis [26]. UCP-1 is mainly expressed in brown adipocytes, along with expression in brite (brown-in-white) or beige adipocytes [27, 28]. Analyzing the effects of PPE on thermogenic gene expressions (*Ucp1*, *Prdm16*, *Pgc1a*, and *Cidea*) in ASC revealed that PPE did not induce thermogenic gene expressions (Additional Fig. 5). Analyzing the effects of PPE on interscapular brown adipose tissue-derived stromal vascular fraction cells revealed that it minimally affected the gene expression of adipocyte differentiation markers (*Pparg*, *Cebpa* and *Adipoq*) or the accumulation of LD, but rather decreased thermogenic gene expressions (Additional Fig. 6). Thus, reducing the accumulation and size of LD in ASC is presumably not a consequence of increase in thermogenic activities.

LAL hydrolyzes cholesteryl ester and triglycerides delivered to the lysosomes into free cholesterol and free fatty acids [29]. Its deficiency causes triglyceride and cholesterol ester accumulation in various body tissues [30]. In contrast, LIPA upregulation increases cytosolic free cholesterol, leading to compensatory transcriptional downregulation of the cholesterol synthesis pathway [31]. Therefore, we examined the effect of PPE on the expression levels of genes in the cholesterol synthetic pathway (*Srebp1*, *Srebp2*, *Hmgcr*, *Fdft1* and *Cyp51*) in ASC-differentiated adipocytes. PPE decreased the expression levels of genes in the cholesterol synthetic pathway, suggesting that PPE increases LIPA expression (Additional Fig. 7). Forkhead homeobox type protein O1 (FoxO1) exerts the transcriptional control of lipid catabolism by inducing *Lipa*, and LAL-mediated degradation of LD has been implicated in lipophagy [32]. Although the effect of PPE on FoxO1 expression is unclear, PPE enhanced *Lipa* expression and autophagosome formation in the ASC-differentiated adipocytes, suggesting that PPE promotes the degradation of LD via lipophagy.

PPE suppresses the differentiation of 3T3-L1 preadipocytes to mature adipocytes via the accelerated activation of p38 MAPK in the early differentiation phase [16]. p38 MAPK, acting upstream of mTORC1, negatively controls autophagy, and p38 MAPK activation inhibits mTORC1 signaling [33]. In contrast, the p38 MAPK pathway may serve as a positive or negative regulator, depending on the cell type, nature of stimulus, and strength and duration of the activated MAPK pathways [34]. The accelerated activation of p38 MAPK may also facilitate PPE-induced lipophagy in ASC. In this regard, we found that PPE did not affect the phosphorylation of extracellular signal-regulated kinase, c-Jun N-terminal kinase, or p38 MAPK in ASC-differentiated adipocytes (Additional Fig. 8). Similarly, PPE did not affect the expression levels of unc-51 like autophagy activating kinase 1 and p70S6K, direct downstream targets of mTORC1, and their phosphorylation state (Additional Fig. 9). Thus, the hypothesis that LD size reduction in ASC-differentiated adipocytes by PPE involves autophagy may be further strengthened by examining the timing of sample collection. Using 3T3-L1 cells, the first 2 days of differentiation induction are crucial in inhibiting adipocyte differentiation by PPE, and a significant increase in the PPE-induced phosphorylation of p38 MAPK is observed particularly 1 h after differentiation induction [16]. This study revealed an increase in the expression of LC3A/B-II by PPE (Fig. 3A), whereas p62 expression did not change significantly, although it tended to decrease (Additional Fig. 10). In addition, *the expressions of LC3A/B-II and p62 significantly increased in cells treated with bafilomycin A1 for 48 h compared to those in bafilomycin A1 non-treated cells*; however, there was no difference between the control and PPE-treated cells. As with the immunoblotting experiment for MAPK signaling, the timing of sample collection and the duration of bafilomycin A1 treatment should be optimized in future.

In conclusion, PPE decreased the size of LD in ASC-differentiated adipocytes. Further, PPE enhanced autophagosome formation and the expression of *Lipa*, suggesting that PPE reduces the accumulation of LD in ASC-differentiated adipocytes through lipophagy. The effects of PPE on other selective autophagy in various types of cells should also be examined, which may suggest potential therapeutic strategies for obesity and propose better utilization of PPE for various diseases.

### Limitations

This study had some limitations that warrant discussion. Primarily, the active substance(s) in the placental extract which induced lipophagy was not identified. In addition, the mechanism of PPE-induced autophagy has not been fully elucidated, and further optimizing the sample collection time in immunoblotting analysis is needed. Moreover, here we used CD31^−^ and CD45^−^ cell populations in eWAT as ASC, based on the previous study. Since the ASC-differentiated adipocytes used in the experiment were a heterogeneous population, the methods for ASC purification and conditions for the differentiation of all ASC into adipocytes require further research. Moreover, only PPE from SNOWDEN was used in this study; validation using PPE from other manufacturers and different animal sources is desired. In addition, the RT-qPCR results should be also be confirmed at the protein level.

### Electronic supplementary material

Below is the link to the electronic supplementary material.

Supplementary Material 1

Supplementary Material 2

Supplementary Material 3

Supplementary Material 4

## Data Availability

The datasets supporting the conclusions of this article are included within the article and its additional files.

## Abbreviations

***ASC***: Adipose-derived mesenchymal stromal/stem cells
***ATP***: Adenosine triphosphate
***C/EBPα***: CCAAT/enhancer binding proteinα
***C/EBPs***: CCAAT/enhancer binding proteins
***DEX***: Dexamethasone
***FBS***: Fetal bovine serum
***FoxO1***: Forkhead homeobox type protein O1
***IBMX***: 3-isobutyl-1-methylxanthine
***LAL***: Lysosomal acid lipase
***LD***: Lipid droplets
***PPARγ***: Peroxisome proliferator-activated receptorγ
***PPE***: Porcine placental extract
***UCP1***: Uncoupling protein-1
