# Supplementary material for: Placental extract suppresses lipid droplet accumulation by autophagy during the differentiation of adipose-derived mesenchymal stromal/stem cells into mature adipocytes
Source: BMC Res Notes. 2023 Nov 16;16:338. doi: 10.1186/s13104-023-06622-6 (PMC10655368; doi:10.1186/s13104-023-06622-6)
Supplement: Supplementary file 3 — Supplementary Material 3 [file 13104_2023_6622_MOESM3_ESM.pdf]

Additional Figure 1

Ando *et al.*

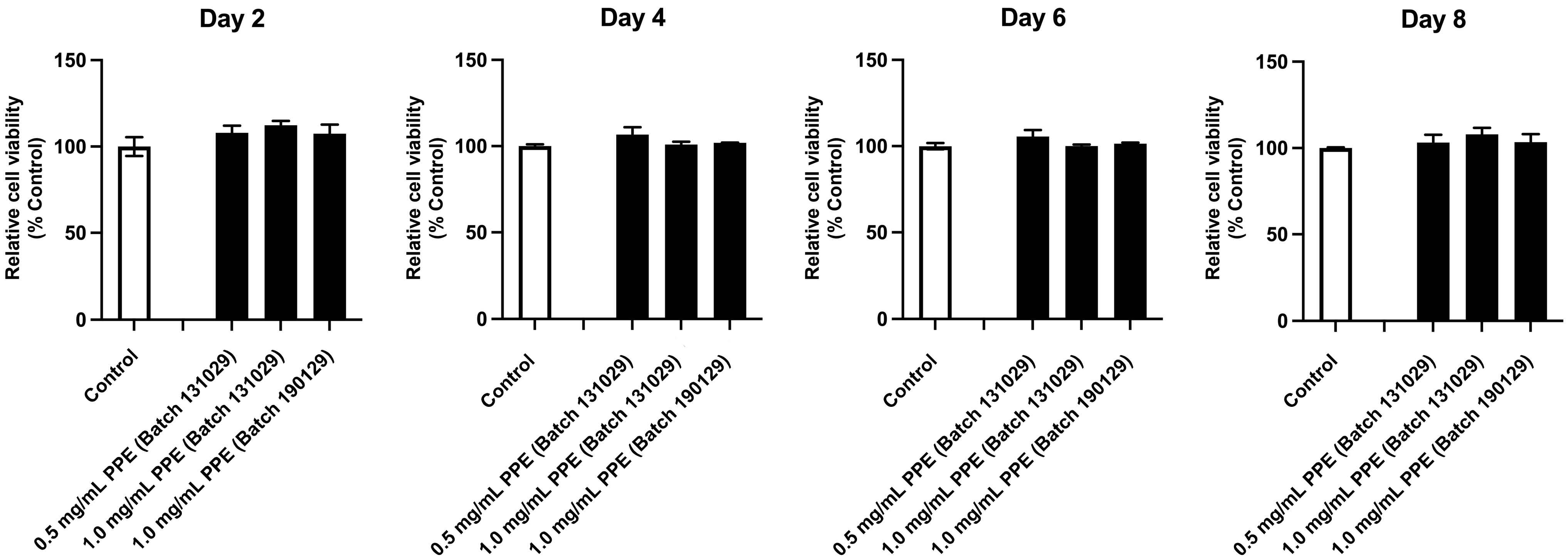

Additional Figure 1 Cytotoxicity of PPE against ASC

ASC were plated in a 96-well plate at a density of 1×10^4 cells/well in complete media. After 24 hours, cells were treated with 1.0 mg/mL of PPE (Batch 131029 used in this study or Batch 190129) for 2, 4, 6 or 8 days. The cytotoxicity of PPE against ASC was assessed using the WST assay (please refer to the Additional Materials and Methods section for details). The data represents relative cell viabilities compared to ASC cultured without PPE (*Control*).

The data are presented as the mean ±SEM.

Additional Figure 2

Ando *et al.*

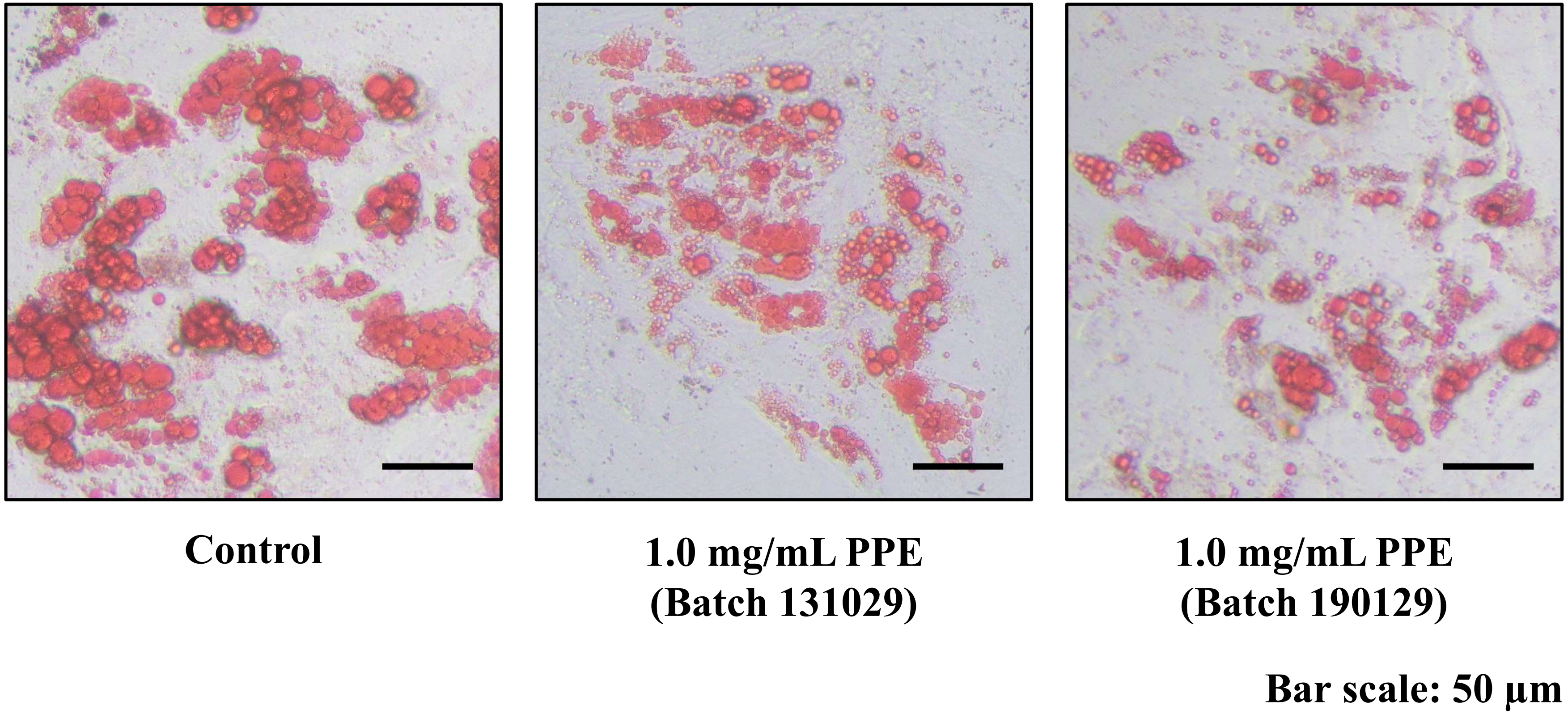

Additional Figure 2 Oil Red O staining for ASC-differentiated adipocytes

Lipid droplets in the ASC cultured for 8 days with or without 1.0 mg/mL PPE (Batch 131029 used in this study or Batch 190129) were stained with Oil Red O and visualized using bright field microscopy.

Additional Figure 3 *Ando et al.*

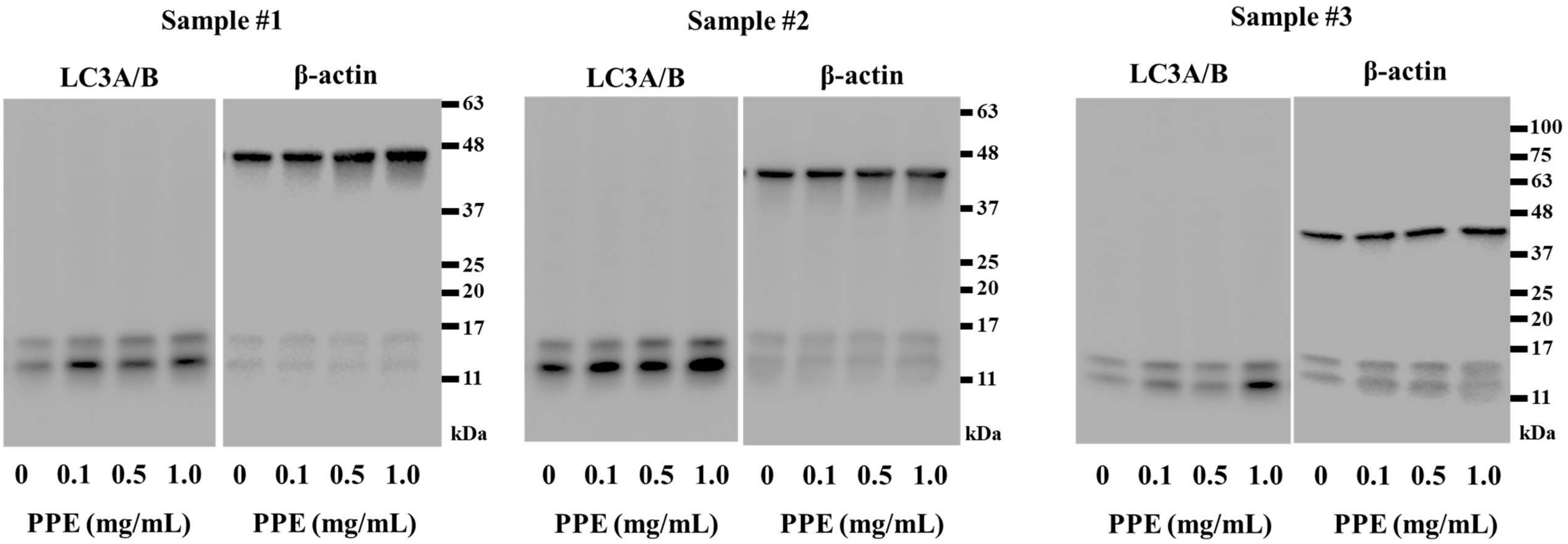

Additional Figure 3 Uncropped immunoblotting images for Figure 3A

After detection of LC3A/B, membranes were stripped and restained with anti-β-actin antibody (loading control). Experiments were performed in triplicate (Sample #1, #2, and #3)

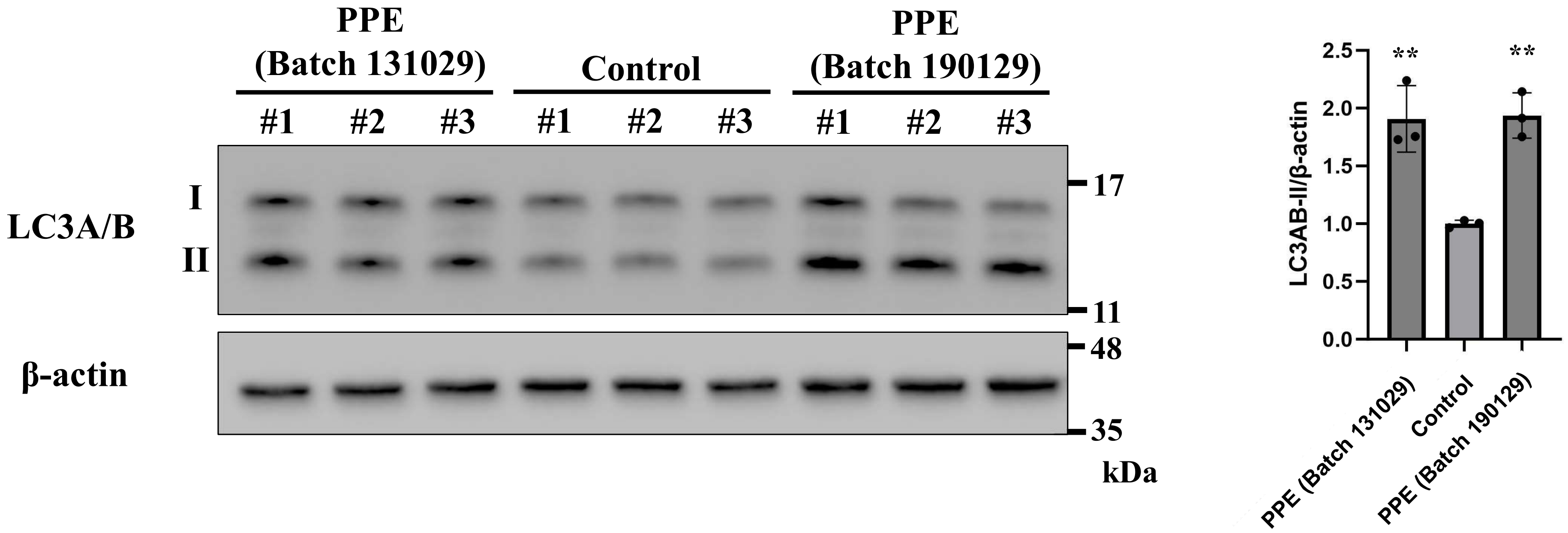

Additional Figure 4 Immunoblotting for LC3A/B in ASC-differentiated adipocytes

Expression levels of LC3A/B in ASC cultured for 8 days with or without 1.0 mg/mL PPE (Batch 131029 used in this study or Batch 190129) were analyzed using immunoblotting. The relative intensity of each LC3A/B-II band after normalization to β-actin levels is shown in the right panel.
The data are presented as the mean ± SEM. **$p < 0.01$ vs. Control

Additional Figure 5

Ando *et al.*

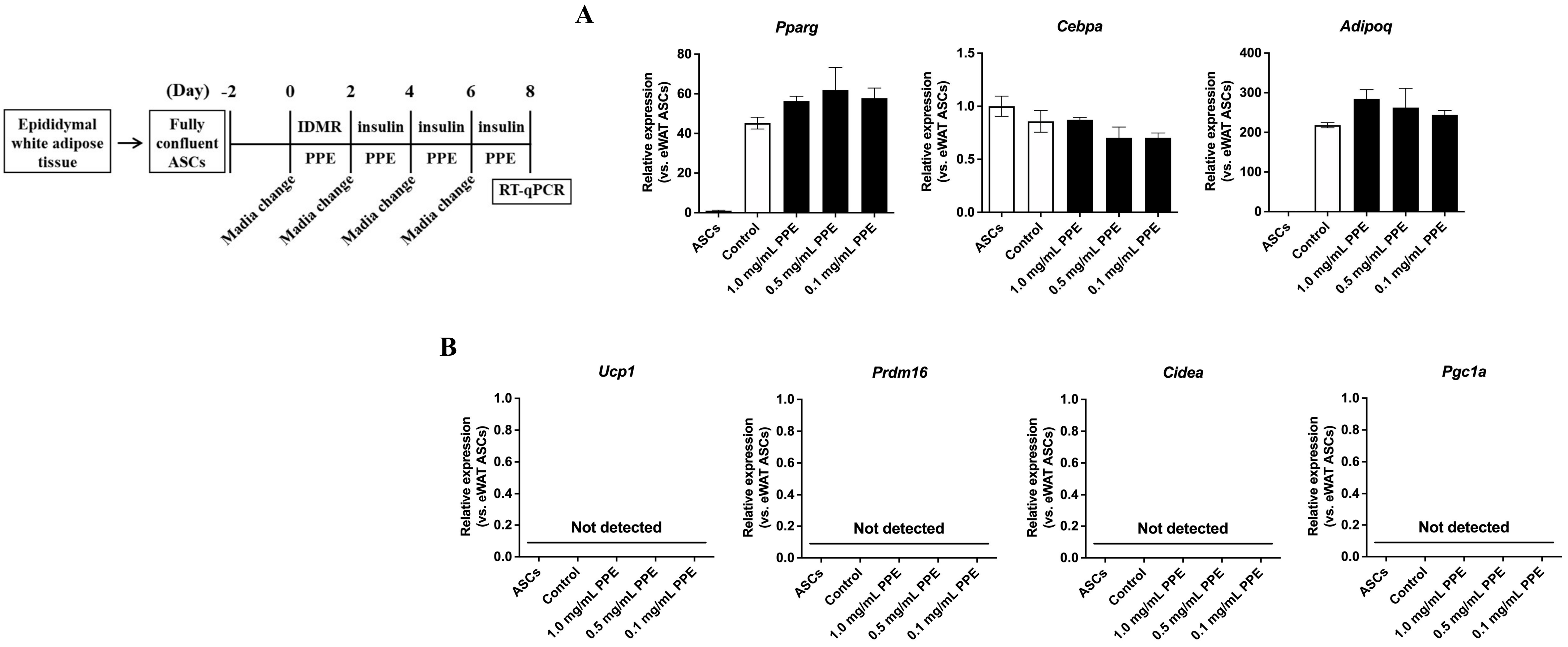

Additional Figure 5 Effect of PPE on thermogenic gene expressions in ASC-differentiated adipocytes

The gene expressions of (A) adipocyte differentiation markers (*Pparg*, *Cebpa*, and *Adipoq*) and (B) thermogenic genes (*Ucp1*, *Prdm16*, *Cidea*, and *Pgc1a*) in ASCs cultured for 8 days with 1.0 mg/mL PPE were analyzed using RT-qPCR. *Control* represents cells cultured without PPE, and *ASCs* represents cells cultured without PPE or differentiation-inducing agents. The gene expressions were presented relative to the value of those from the *ACSs*. All RT-qPCR experiments were performed in triplicate, and the data are presented as the mean ± SEM.

Additional Figure 6

Ando *et al.*

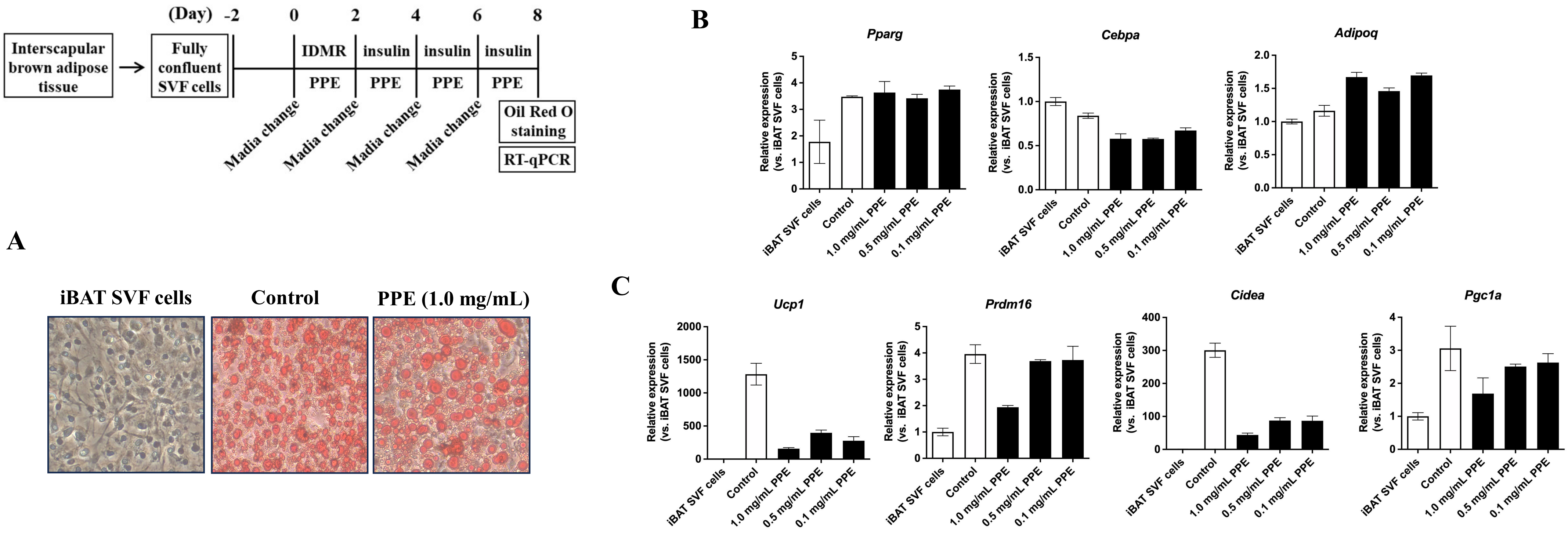

Additional Figure 6 Effect of PPE on adipocyte differentiation markers and thermogenic gene expressions in interscapular brown adipose tissue (iBAT)-derived stromal vascular fraction (SVF) cells

(A) Lipid-droplet in iBAT SVF cells cultured for 8 days with or without 1.0 mg/mL PPE were stained with Oil Red O and visualized using bright field microscopy. (B) and (C) The gene expressions of (A) adipocyte differentiation markers (*Pparg*, *Cebpa*, and *Adipoq*) and (B) thermogenic genes (*Ucp1*, *Prdm16*, *Cidea*, and *Pgc1a*) in iBAT SVF cells cultured for 8 days with 1.0 mg/mL PPE were analyzed using RT-qPCR. *Control* represents cells cultured without PPE, and *iBAT SVF cells* represents the cells cultured without PPE or differentiation-inducing agents. The gene expressions were presented relative to the value of those from the *iBAT SVF cells*. All RT-qPCR experiments were performed in triplicate, and the data are presented as the mean ± SEM.

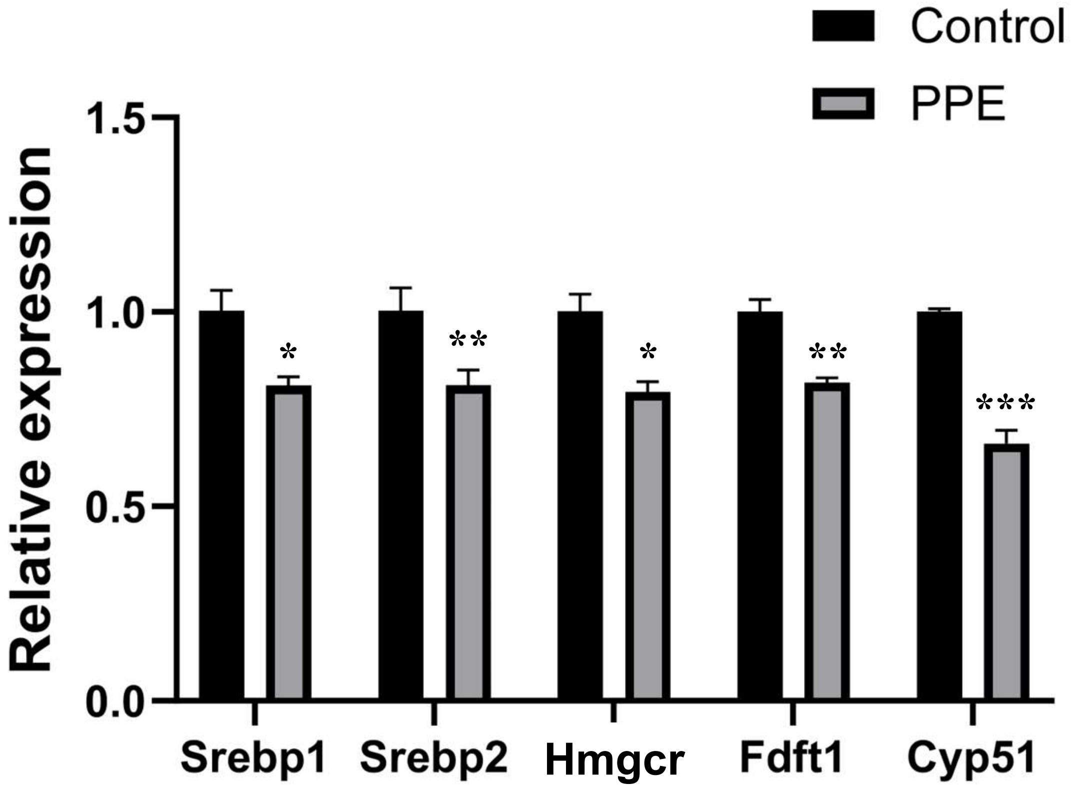

Additional Figure 7 RT-qPCR analysis for the expression levels of genes in the cholesterol synthetic pathway

The gene expressions in the cholesterol synthetic pathway (*Srebp1*, *Srebp2*, *Hmgcr*, *Fdft1* and *Cyp51*) in ASC cultured for 8 days with 1.0 mg/mL PPE were analyzed using RT-qPCR. *Control* represents cells cultured without PPE. The expression of each genes is presented relative to the value in *Control*. All RT-qPCR experiments were performed in triplicate, and the data are presented as the mean ± SEM. *p < 0.05, **p < 0.01 , ***p < 0.001 (vs. Control.)

Additional Figure 8

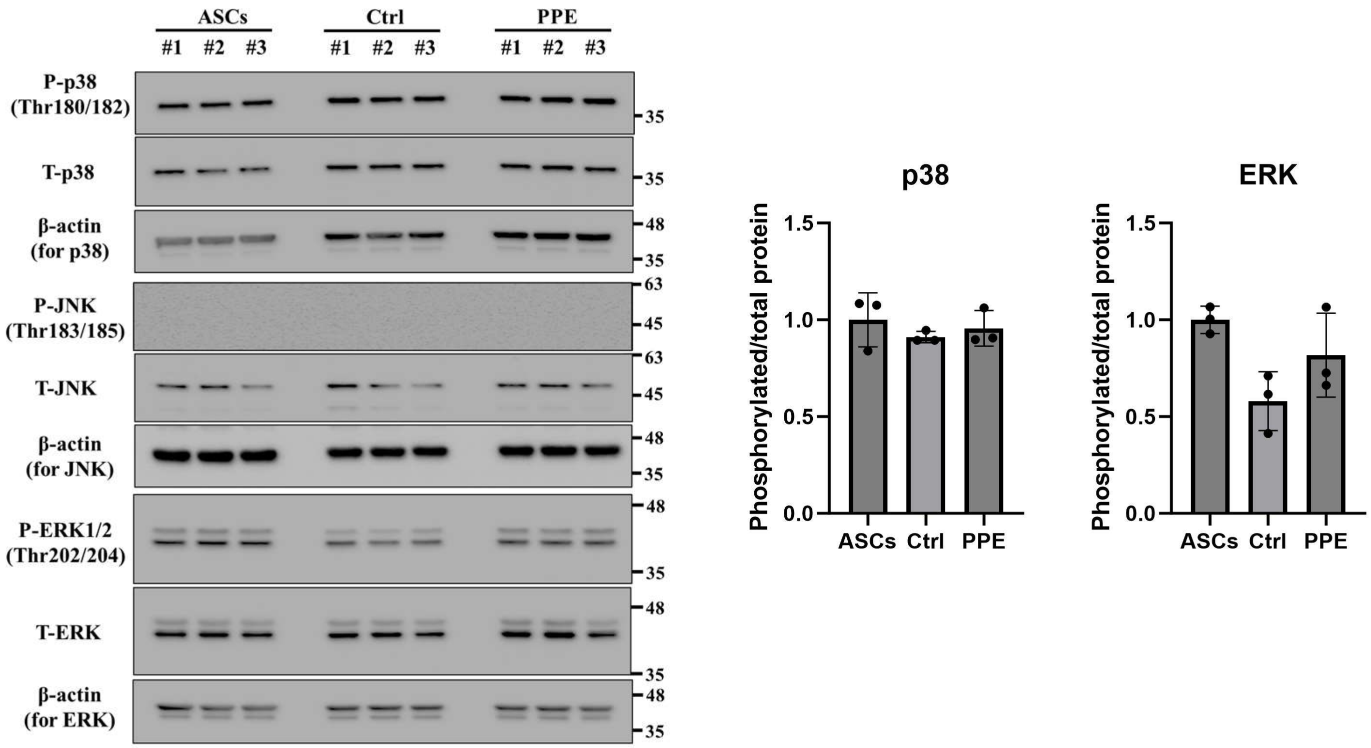

Additional Figure 8 Immunoblotting for MAPK signaling in ASC-differentiated adipocytes

The phosphorylation state of p38, JNK, and ERK1/2 in ASC cultured for 8 days with or without 1.0 mg/mL PPE were analyzed using immunoblotting. The relative intensity of phosphorylated protein to total protein levels are shown in the right panel. *ASC* represents cells cultured without PPE or differentiation-inducing agents. The data are presented as the mean ± SEM.

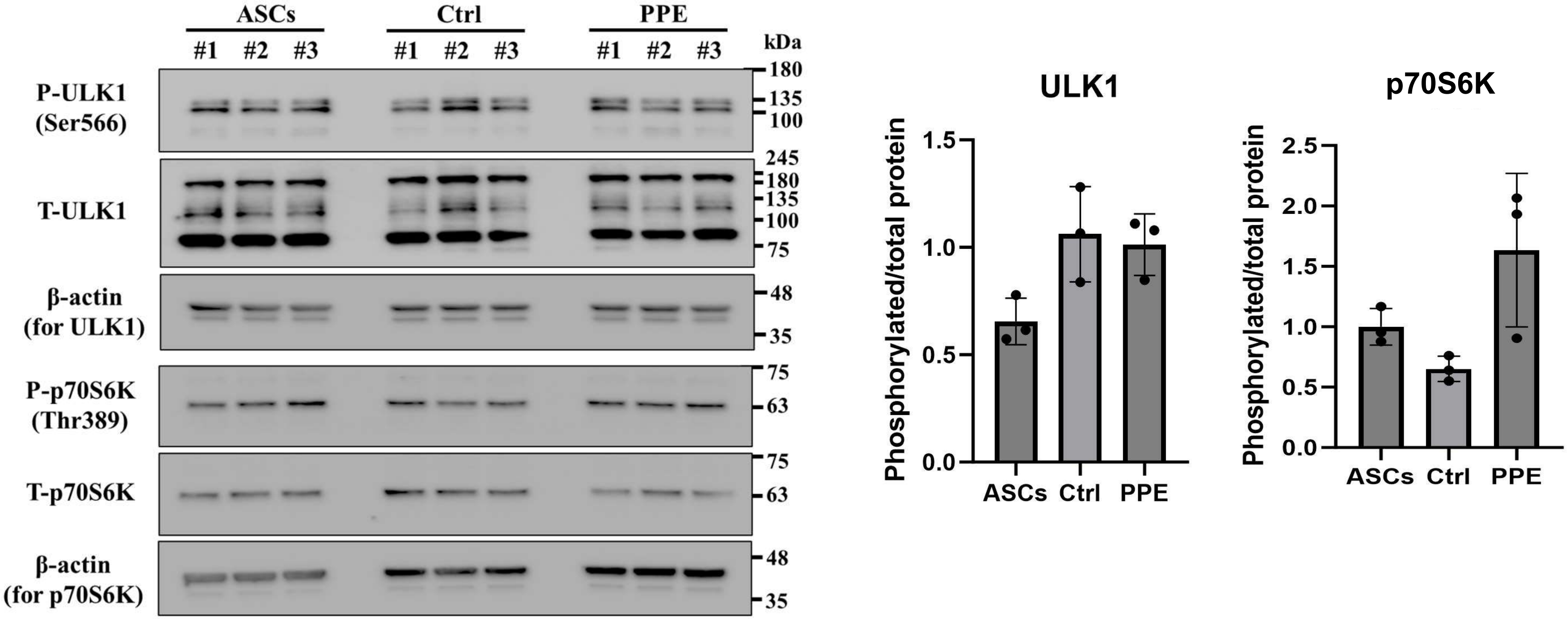

Additional Figure 9 Immunoblotting for ULK1 and p70S6K in ASC-differentiated adipocytes

The phosphorylation state of ULK1 and p70S6K in ASC cultured for 8 days with or without 1.0 mg/mL PPE were analyzed using immunoblotting. The relative intensity of phosphorylated protein to total protein levels are shown in the right panel. *ASC* represents cells cultured without PPE or differentiation-inducing agents. The data are presented as the mean ± SEM.

Additional Figure 10

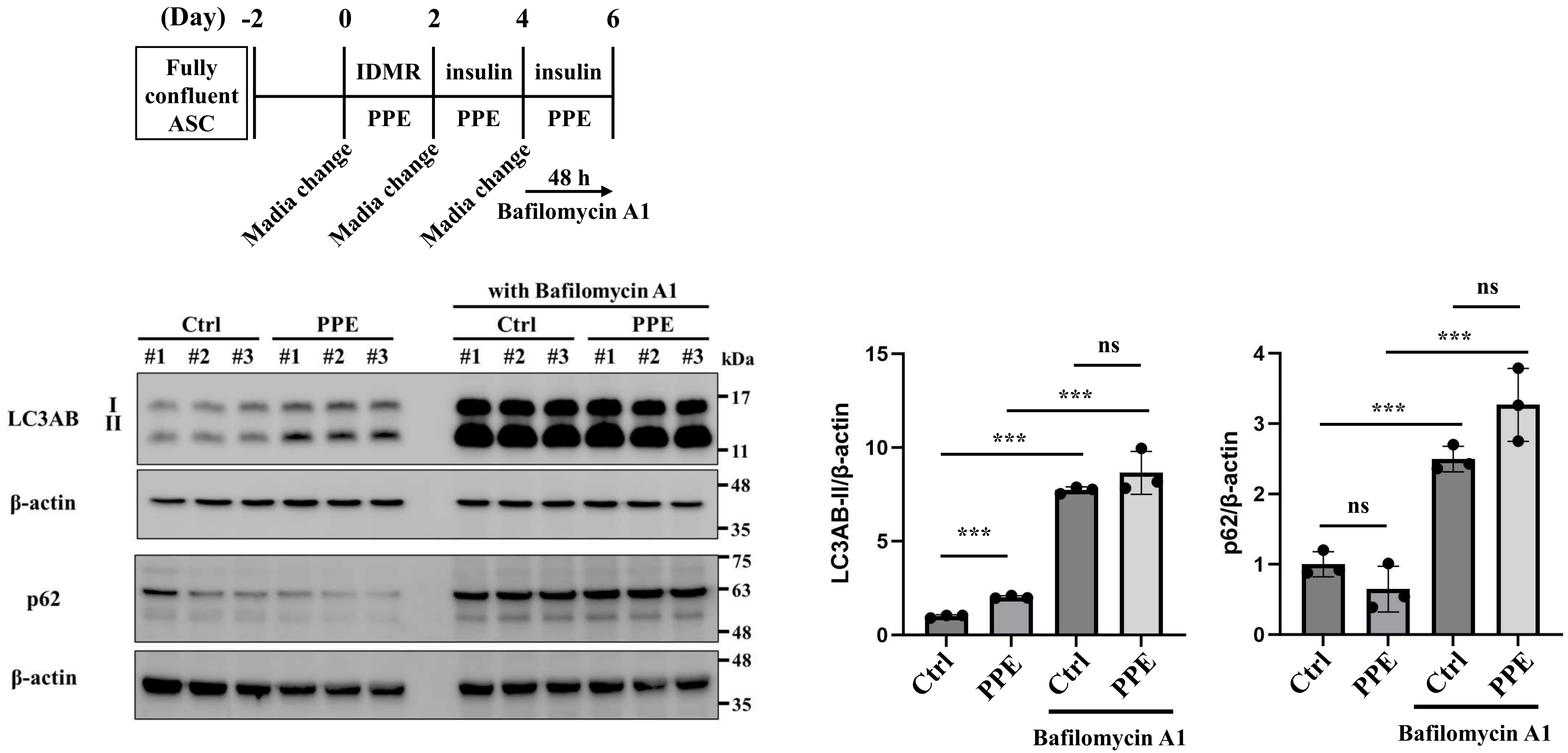

Additional Figure 10 Immunoblotting for LC3A/B and p62 in ASC-differentiated adipocytes treated with Bafilomycin A1

ASC were cultured for 4 days with or without 1.0 mg/mL of PPE, and subsequently treated with Bafilomycin A1 for an additional 48 hours. Expression levels of LC3A/B and p62 were analyzed using immunoblotting. The relative intensity of each LC3A/B-II and p62 band, normalized to β-actin levels, is displayed in the right panel.
The data are presented as the mean ± SEM. ***$p < 0.001$, ns: not significant."
