# Supplementary material for: Placental extract suppresses lipid droplet accumulation by autophagy during the differentiation of adipose-derived mesenchymal stromal/stem cells into mature adipocytes
Source: BMC Res Notes. 2023 Nov 16;16:338. doi: 10.1186/s13104-023-06622-6 (PMC10655368; doi:10.1186/s13104-023-06622-6)
Supplement: Supplementary file 4 — Supplementary Material 4 [file 13104_2023_6622_MOESM4_ESM.docx]

**Additional** **Materials and Methods**

***Preparation of adipose-derived mesenchymal stromal/stem cells (ASC) and*** *interscapular brown adipose tissue-derived stromal vascular fraction cells (iBAT)*

Epididymal white adipose tissue (eWAT) or interscapular brown adipose tissue (iBAT) of male BALB/c mice (7 weeks, Japan SLC, Inc., Shizuoka, Japan) was collected and cut into 1 mm^3^ pieces, followed by dissociating at 37 °C for 1 hour in D-MEM/Ham's F-12 medium containing 2 mg/mL Type-IV Collagenase (Thermo Fisher Scientific, MA, USA). After filtrating through a 40 µm Cell Strainer (BD Biosciences, NJ, USA) and centrifuged at 1,000 × g for 5 min, the cell pellet was treated with ACK lysing buffer (Thermo Fisher Scientific) to lyse the red blood cells. The biotin-labeled anti-mouse CD31 and CD45 antibodies (BioLegend, CA, USA) were added to the cells and incubated at 4°C for 15 min, followed by the addition of MojoSort Streptavidin Nanobeads (BioLegend). After incubation at 4°C for 15 min, the magnetically labeled cells were removed using a MojoSort magnet (BioLegend). The untouched fraction was collected and used as adipose-derived mesenchymal stromal/stem cells (ASC) or iBAT stromal vascular fraction (SVF) cells in this study. All procedures for the experiments using mice were conducted in accordance with the Guide for Care and Use of Laboratory Animals of Josai University. The animal protocols were approved by the Animal Care and Use Committee of Josai University (protocol: JU22071). All procedures were performed under anesthesia and were designed to minimize suffering.

***WST assay***

The cytotoxicity of PPE against ASC was evaluated by WST assay using a Cell Counting Kit-8 (Dojindo Laboratories, Kumamoto, Japan). ASC were plated on a 96-well plate at a density of 1 × 10^4^ cells/well in complete media. After 24h, cells were treated with PPE at various concentrations for a further 48h. The procedures were performed according to the manufacturer's protocols.

***Immunoblotting***

Cells were lysed in a 1 × SDS sample buffer (50 mM Tris-HCl, 1% SDS, 5% glycerol, 0.01% bromophenol blue, pH 6.8). The cell lysates were sonicated and subjected to SDS-PAGE followed by immunoblotting as previously described [16].
